# The Aging Muscle in Experimental Bed Rest: A Systematic Review and Meta-Analysis

**DOI:** 10.3389/fnut.2021.633987

**Published:** 2021-08-04

**Authors:** Filippo Giorgio Di Girolamo, Nicola Fiotti, Zoran Milanović, Roberta Situlin, Filippo Mearelli, Pierandrea Vinci, Boštjan Šimunič, Rado Pišot, Marco Narici, Gianni Biolo

**Affiliations:** ^1^Clinica Medica, Azienda Sanitaria Universitaria Giuliano Isontina, Department of Medical, Surgical and Health Sciences, University of Trieste, Trieste, Italy; ^2^SC Assistenza Farmaceutica, Azienda Sanitaria Universitaria Giuliano Isontina, Department of Medical, Surgical and Health Sciences, University of Trieste, Trieste, Italy; ^3^Faculty of Sport and Physical Education, University of Niš, Niš, Serbia; ^4^Science and Research Centre Koper, Institute for Kinesiology Research, Koper, Slovenia; ^5^Faculty of Sports Studies, Incubator of Kinanthropological Research, Masaryk University, Brno, Czechia; ^6^Department of Biomedical Sciences, Neuromuscular Physiology Laboratory, University of Padova, Padova, Italy

**Keywords:** bed rest, aging, muscle mass, muscle physiopathology, muscle function

## Abstract

**Background:** Maintaining skeletal muscle mass and function in aging is crucial for preserving the quality of life and health. An experimental bed rest (BR) protocol is a suitable model to explore muscle decline on aging during inactivity.

**Objective:** The purpose of this systematic review and meta-analysis was, therefore, to carry out an up-to-date evaluation of bed rest, with a specific focus on the magnitude of effects on muscle mass, strength, power, and functional capacity changes as well as the mechanisms, molecules, and pathways involved in muscle decay.

**Design:** This was a systematic review and meta-analysis study.

**Data sources:** We used PubMed, Medline; Web of Science, Google Scholar, and the Cochrane library, all of which were searched prior to April 23, 2020. A manual search was performed to cover bed rest experimental protocols using the following key terms, either singly or in combination: “Elderly Bed rest,” “Older Bed rest,” “Old Bed rest,” “Aging Bed rest,” “Aging Bed rest,” “Bed-rest,” and “Bedrest”. Eligibility criteria for selecting studies: The inclusion criteria were divided into four sections: type of study, participants, interventions, and outcome measures. The primary outcome measures were: body mass index, fat mass, fat-free mass, leg lean mass, cross-sectional area, knee extension power, cytokine pattern, IGF signaling biomarkers, FOXO signaling biomarkers, mitochondrial modulation biomarkers, and muscle protein kinetics biomarkers.

**Results:** A total of 25 studies were included in the qualitative synthesis, while 17 of them were included in the meta-analysis. In total, 118 healthy elderly volunteers underwent 5-, 7-, 10-, or 14-days of BR and provided a brief sketch on the possible mechanisms involved. In the very early phase of BR, important changes occurred in the skeletal muscle, with significant loss of performance associated with a lesser grade reduction of the total body and muscle mass. Meta-analysis of the effect of bed rest on total body mass was determined to be small but statistically significant (ES = −0.45, 95% CI: −0.72 to −0.19, *P* < 0.001). Moderate, statistically significant effects were observed for total lean body mass (ES = −0.67, 95% CI: −0.95 to −0.40, *P* < 0.001) after bed rest intervention. Overall, total lean body mass was decreased by 1.5 kg, while there was no relationship between bed rest duration and outcomes (*Z* = 0.423, *p* = 672). The meta-analyzed effect showed that bed rest produced large, statistically significant, effects (ES = −1.06, 95% CI: −1.37 to −0.75, *P* < 0.001) in terms of the knee extension power. Knee extension power was decreased by 14.65 N/s. In contrast, to other measures, meta-regression showed a significant relationship between bed rest duration and knee extension power (*Z* = 4.219, *p* < 0.001). Moderate, statistically significant, effects were observed after bed rest intervention for leg muscle mass in both old (ES = −0.68, 95% CI: −0.96 to −0.40, *P* < 0.001) and young (ES = −0.51, 95% CI: −0.80 to −0.22, *P* < 0.001) adults. However, the magnitude of change was higher in older (MD = −0.86 kg) compared to younger (MD = −0.24 kg) adults.

**Conclusion:** Experimental BR is a suitable model to explore the detrimental effects of inactivity in young adults, old adults, and hospitalized people. Changes in muscle mass and function are the two most investigated variables, and they allow for a consistent trend in the BR-induced changes. Mechanisms underlying the greater loss of muscle mass and function in aging, following inactivity, need to be thoroughly investigated.

## Introduction

Preserving skeletal muscle mass and strength throughout the lifespan is recognized as a primary factor to maintain an adequate quality of life and survival. After the age of 50, about 0.5–1% of muscle mass can be lost, even after considering inter-individual genetic and lifestyle differences (1). This para-pathological status, called sarcopenia of aging, can worsen the quality of life and lead to premature death (2). Recently, an increase in the so-called “intrinsic capacity” (i.e., the composite combination of physical and mental capacities of an individual) has been identified as the main step to promote healthy aging (3). As a result, physical inactivity, to the extreme of being confined to bed, is a key factor in contributing to the onset of functional ability decline in the elderly (4). Several studies demonstrate that, especially in the elderly, physical inactivity increases the risk of fractures, due to falling (5), and worsens their general health conditions including protein and glucose metabolism (6), cardiovascular function (7), and cognitive capacity (8). Inactivity also seems to exacerbate motor unit denervation associated with aging (9) and neuromuscular junction degeneration (NMJ) (10). On the contrary, an active lifestyle seems to protect against these neurodegenerative changes associated with aging and inactivity (11).

In this perspective, the negative health effect of hospitalization (or institutionalization) could be, at least partially, explained by the sudden reduction in physical activity (12) causing muscle loss not only through unloading *per se* but also by triggering muscle denervation (13, 14) and NMJ damage (15, 16). NMJ damage is considered a key driver of sarcopenia (17). Recent studies consider physical inactivity (or bed rest, BR) during hospitalization, as a primary factor contributing to the functional and cognitive decline of older patients (8). Hospitalized patients, including those able to walk independently, spend most of their time in bed (18). This is an important factor in healthcare planning as most hospitalized patients are elderly and their prevalence is expected to double by 2030 (19). In fact, 75% of hospitalized elderly patients stand or walk only for an average of 5.5 min/day (20) and, in general, for no more than 5% of the entire daytime. Moreover, during hospitalization, their ambulatory function and daily living activities decline by 30–55 and 65%, respectively (21–26). This often leads to the institutionalization in a nursing home after discharge (27). Almost 50% of elderly patients hospitalized for a non-disabling condition suffer a long-term (1 month or more) functional decline at discharge (28). In addition, lower muscle mass and strength are more likely to prolong the length of stay, increase the risk of readmission after discharge, and eventually boost the hospitalization costs (29).

Due to these clinical implications, the effects of physical inactivity in older participants (and the possibility to prevent and/or counteract them) have been widely investigated in the past years utilizing different approaches and research protocols. Experimental BR is one of the most suitable approaches in studying consequences of physical inactivity in a controlled, standardized and realistic environment.

Investigators have recognized the potential clinical relevance of the BR model to mimic the physical inactivity experienced during hospitalization, illness, and injury while allowing to differentiate the catabolic, disease-related effects from the intrinsic consequences of skeletal muscle disuse. A small number of experimental immobilization studies have been carried out in older volunteers during the last 10 years (30–49). Considering the risk of thromboembolic complications (50–52) in older subjects, less hazardous, alternative approaches, such as unilateral leg immobilization (50), unilateral leg suspension (51), or step reduction (53), have been recommended. Although these approaches are very useful in the assessment of muscle physiology or single muscle fiber properties, they are not suitable for the investigation of the metabolic and systemic changes occurring during prolonged inactivity or BR (46), which are allegedly responsible for the reduced survival rate. The use of specific precautions, like compression leg stockings and/or strict monitoring of coagulation parameter (i.e., D-Dimer levels) (46), to prevent thromboembolic events could increase trust in the BR approach, which so far, is considered as a valid approach to explore the effects of physical inactivity in humans. Experimental BR in older participants provides, therefore, unique results, and the present systematic review and meta-analysis focuses on clinical metabolic and pathophysiological events occurring during 5, 7, 10, and 14 days of BR.

This systematic review and meta-analysis evaluates the published studies on the effects of experimental bed rest on muscle mass and function in aging populations as compared to young controls. Moreover, this work also focuses on the different effects of rehabilitation protocols both in young and old subjects. This is an emerging topic. In fact, physical inactivity and immobilization have a great clinical impact on health and disease progression, and rehabilitation therapies are not frequently and efficiently implemented. Such scenarios dramatically worsened during the covid-19 pandemic (54).

## Materials and Methods

### Search Strategy and Study Selection

The systematic review and meta-analysis were performed in accordance with the Preferred Reporting Items for Systematic Reviews and Meta-Analyses (PRISMA) guidelines (55).

Computerized literature searches were conducted for articles in the following electronic databases: PubMED, Medline; Web of Science, Google Scholar, and Cochrane library. The search strategy was designed in PubMED and subsequently applied to Cochrane library, Medline, Web of Science, and Google Scholar. The final reference lists of the included studies were reviewed for additional relevant studies. A structured search included papers published prior to March 1, 2021. A manual database search was performed using the following key terms, either singly or in combination: “Elderly Bed rest” and “Bed-rest” and “Bedrest”; OR “Older Bed rest” and “Bed-rest” and “Bedrest”; OR “Old Bed rest” and “Bed-rest” and “Bedrest”; OR “Aging Bed rest” and “Bed-rest” and “Bedrest”; OR “Aging Bed rest” and “Bed-rest” and “Bedrest.”

**Figure 1:**
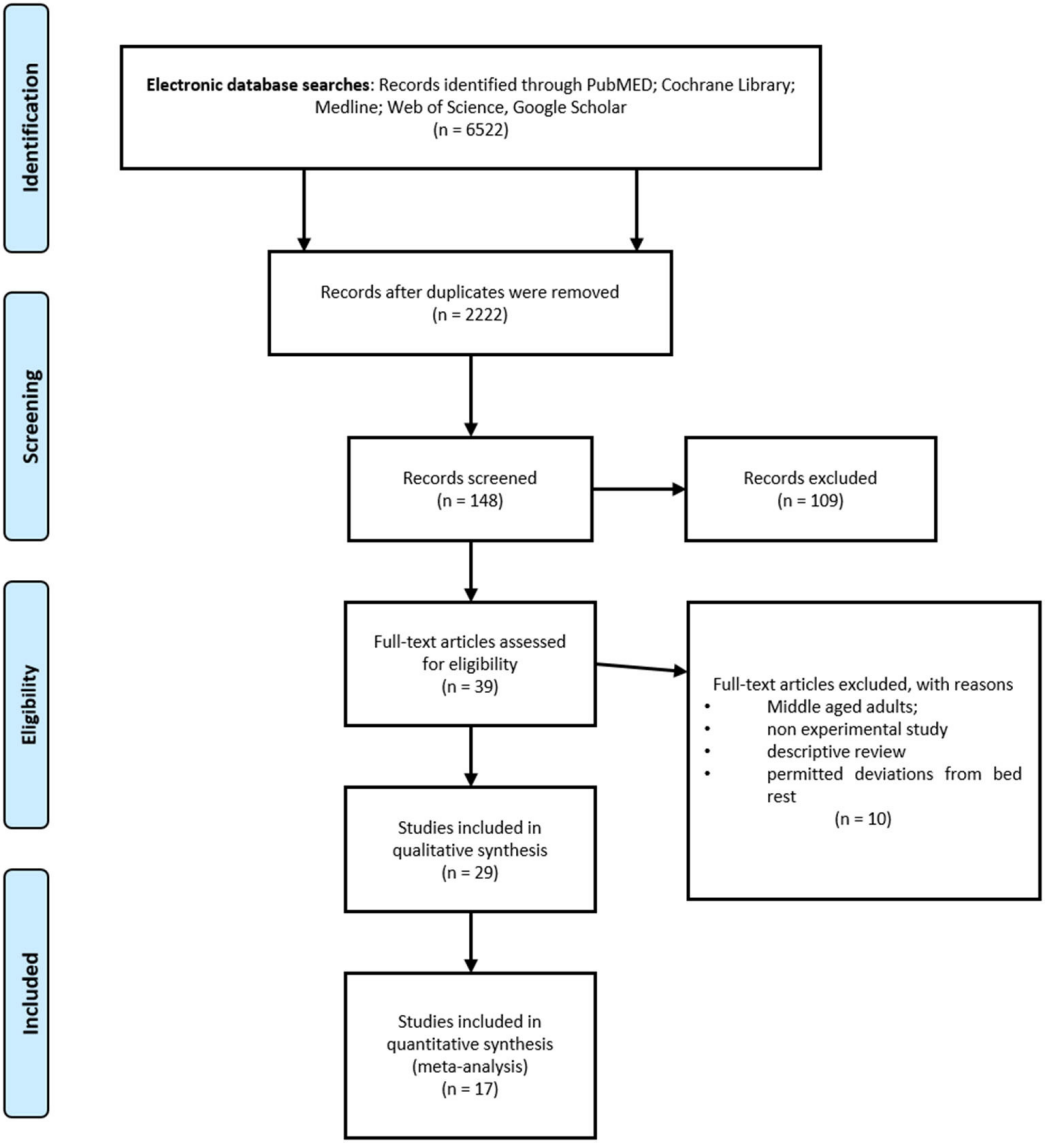
PRISMA flow diagram.

The study selection process is presented in Figure 1. Initially, two independent reviewers performed the following steps: literature search, identification, screening, quality assessment, and data extraction. First, all titles were screened during database searches to assess the suitability of publications. After that, abstracts were screened using predetermined inclusion and exclusion criteria. Then, full texts were reviewed by the two reviewers to reach a final decision. Apart from those steps, reference lists from retrieved papers were examined for additional potentially eligible papers. Consensus or arbitration by a third reviewer were used in cases of any disagreement between reviewers.

The Cochrane risk of bias assessment, including the following items random sequence generation, allocation concealment, blinding of participants and personnel, blinding of outcome assessment, incomplete outcome data, selective reporting, and other bias, was used.

Inclusion criteria were as follows: the PICO (participants, intervention, comparison, and outcomes) standard was followed for inclusion and exclusion criteria. The type of participants were healthy adults (>60 years) included in systematic review and meta-analysis. No publication data or publication status restrictions were imposed. Types of interventions were bed rest experimental protocols, which should not allow any deviation from lying in bed and had to last at least 1 day, with participants assigned to either the experimental or the control group. Types of outcome measure were that the primary outcome measures for systematic review and meta-analysis were as follows: body mass index, fat mass, fat-free mass, lower limb lean mass, cross-sectional area, knee extension power, cytokine pattern, IGF signaling biomarkers, FOXO signaling biomarkers, mitochondrial modulation biomarkers, and muscle protein kinetics biomarkers.

Exclusion criteria were as follows: non-experimental studies (i.e., hospital stay), studies written in languages other than English, and duplication publications. These were excluded. Studies with additional interventions, unbalanced diets, or missing relevant data necessary for meta-analysis were also excluded from the analysis.

### Data Extraction

Standardized protocol for data extraction was used to extract: (I) study characteristics with appropriate information regarding author(s), title and year of publication; (II) participant information, such as sample size, age, health status and gender; (III) a description of the bed rest experimental protocols, duration and frequency; (IV) study outcomes including the following measurements: Body Mass Index; Fat Mass; Fat-Free Mass; Leg Lean Mass; Cross Sectional Area; Knee extension power; Cytokine pattern; IGF signaling biomarkers; FOXO Signaling biomarkers; Mitochondrial modulation biomarkers; and Muscle protein kinetics biomarkers. When numeric data were not reported in the text, they were extracted from charts and figures using Graph digitizer software (DigitizeIt, Germany). In most of the studies, mean and SD were reported, while correlation was not: in these instances, the correlation value was set at 0.5, as previously suggested. Data extraction from the included studies was performed independently by two reviewers (N.F. and B.Š.) and checked for accuracy and completeness. Disagreements were resolved by consensus. The entire procedure was not blinded to authors, journals or institutions.

### Quality of the Included Studies

Several tools are available to estimate the quality of randomized, controlled (56), and non-randomized trials or observational studies (57). Our strategy was to include all randomized and non-randomized, observational and interventional studies depending on the risk of confounding internal validity. Each included study has been scored based on the following criteria:

(1) confinement at the test facilities before bed rest (controlled ambulatory restriction); (2) controlled energy intake diet during bed rest; (3) controlled macronutrient intake during bed rest; (4) clearly defined restrictions and permitted deviations from bed rest; (5) clearly defined and objective criteria for the assessment of Body mass measurement; (6) clearly defined and objective criteria for the assessment of Body composition (FFM, FM) measurement; and (7) clearly defined and objective criteria for the assessment of muscle performance.

### Statistical Analysis

Data has been collected from the published versions and online supplementary materials of the manuscript and the effects of BR have been reported by absolute difference or percent change between baseline and end of BR values. Correlation between these changes and the duration of BR has been calculated by Spearman's Rank Correlation. Statistical significance, correlation coefficient, and the best fitting linear association equation have been obtained by SPSS (Statistical Package for Social Sciences, IL, version 21.0). Comprehensive Meta-analysis V.2 software (Biostat, Englewood, New Jersey, USA) was used for the meta-analyses. The standardized difference in means (SDM) and mean difference (MD) with appropriate 95% confidence intervals (CIs) were calculated for all outcome measures. Publication bias was checked using Egger's test and asymmetry of funnel plots. Significant bias was noted if *p* < 0.10. The *I*^2^ statistic was used to investigate between-study heterogeneity; where values of 25, 50, and 75% represent low, moderate, and high statistical heterogeneity, respectively (45). Pooled estimates of the effect of BR on total body mass, total fat body mass, total lean body mass, knee extension power, and leg muscle mass using effect size (ES), were obtained using fixed (*I*^2^ <75%) or random (*I*^2^ > 75%) effects models. ES was classified as: very small (<0.20), small (0.21–0.50), moderate (0.51–0.80), large (0.81–1.20), very large (1.21–2.00), and extremely large (>2.01) (58). Furthermore, meta-regression was performed to examine whether the effects of BR duration on body composition and skeletal muscle performance. Significance was set at *p* < 0.05.

## Results

### Study Selection

The literature search yielded 6,527 studies. During the study selection process, 4,300 duplicates were removed and 2,222 unique study reports remained for title and abstract screening. Following the initial screening of titles, 148 full-text articles were retrieved and assessed for eligibility. The screening of abstracts excluded 109 records. A total of 39 studies met the inclusion criteria. However, the full-text examination identified further 10 studies not meeting the inclusion criteria. A total of 29 studies were included in the qualitative synthesis, while 17 of them were included in the meta-analysis. The PRISMA flow diagram is shown in Figure 1.

### Study Characteristics: Bed Rest Protocols and List of Publications

Nine BR protocols have been conducted in elderly volunteers yielding a total of 29 different publications (30–49). All the protocols were consistent in volunteer recruitment, exclusion criteria, immobilization design, nurse monitoring, and caloric intake, while countermeasures during BR, rehabilitation procedures, and the presence of a control group were heterogeneous.

The studies published so far lasted 5, 7, 10, or 14 days, with the most frequent BR duration of 10 days. Data is available for pre- and post-BR, without intermediate evaluations (Table 1).

**Table 1:** Experimental bed rest protocols involving healthy elderly participants.

	**Study group**				**Comparison group**			
**References**	**Days of bed rest**	**No. of volunteers**	**Age (years)**	**BMI (kg/m^**2**^)**	**No. of volunteers**	**Age (years)**	**BMI (kg/m^**2**^)**	**Rehabilitation program**
**Bed rest protocols**								
Reidy et al. (26) Reidy et al. (27)	5	10	69 ± 2	25.3 ± 1	NMES+PRO (10)	70 ± 2	25.7 ± 0.8	NA
Tanner et al. (29) Reidy et al. (30)	5	9	66 ± 1	25.0 ± 1.0	Young (14)	23 ± 1	22.0 ± 1	8 weeks: resistance exercise + BCAA protein supplementation
Arentson-Lantz et al. (32)	7	10	68 ± 2	25.2 ± 0.7	2,000 steps/day (7)	68 ± 2	27.1 ± 1.4	NA
Arentson-Lantz et al. (33)	7	10	68 ± 2	25.2 ± 0.7	WHEY (5)	69 ± 1	27.4 ± 0.8	5-day rehabilitation
Arentson-Lantz et al. (34)	7	10	68 ± 2	25.2 ± 0.7	Leucine (7)	68 ± 1	28.0 ± 1.0	NA
Drummond et al. (30) Drummond et al. (31)	7	6	67 ± 2	24.7 ± 0.9	No (–)	–	–	NA
Kortebein et al. (23) Kortebein et al. (36)	10	12	67 ± 5	29.0 ± 3.0	No (–)	–	–	NA
Coker et al. (37)	10	8	64+3	28.1 ± 1.7	No (-)	–	–	NA
Ferrando et al. (35)	10	11	68 ± 5	**	EAA (10)	71 ± 6	$$	NA
Deutz et al. (38) Standley et al. (39) Standley et al. (40)	10	8	67 ± 1	26.5 ± 1.2	HMB (11)	67 ± 1	24.9 ± 1.0	8 weeks: strength training + placebo or HMB supplementation
Jurdana et al. (43) Pišot et al. (42) Rejc et al. (44) Buso et al. (45)	14	16	60 ± 3	26.6 ± 4.4	Young (7)	23 ± 3	24.0 ± 2.4	2 weeks: resistance training
Biolo et al. (41)	14	8	60 ± 3	26.8+4.2	Young (7)	23.1+2.9	24+2.4	NA

*Table reports main data of the volunteers included in the bed rest protocols. Study group: Healthy aging volunteers performing a bed rest protocol without any intervention. Comparison group: healthy volunteers performing a bed rest protocol either elderly people having a countermeasure or younger counterparts*.

The “Rehabilitation program” column reports “Not applicable (NA)” when the article stated that a rehabilitation protocol was conducted but no data were reported. ^**^83 ± 19 (kg). We report here body weight of the volunteers since neither body height nor body mass index (BMI) was available. ^$$^72 ± 8 (kg). We report here the body weight of the volunteers since neither body height nor body mass index (BMI) was available.

### Meta-Analysis

Based on Egger's test and funnel plot asymmetry, all observed measures indicated publications bias (*p* < 0.10).

#### Effects of Bed Rest on Body Composition

**Figure 2:**
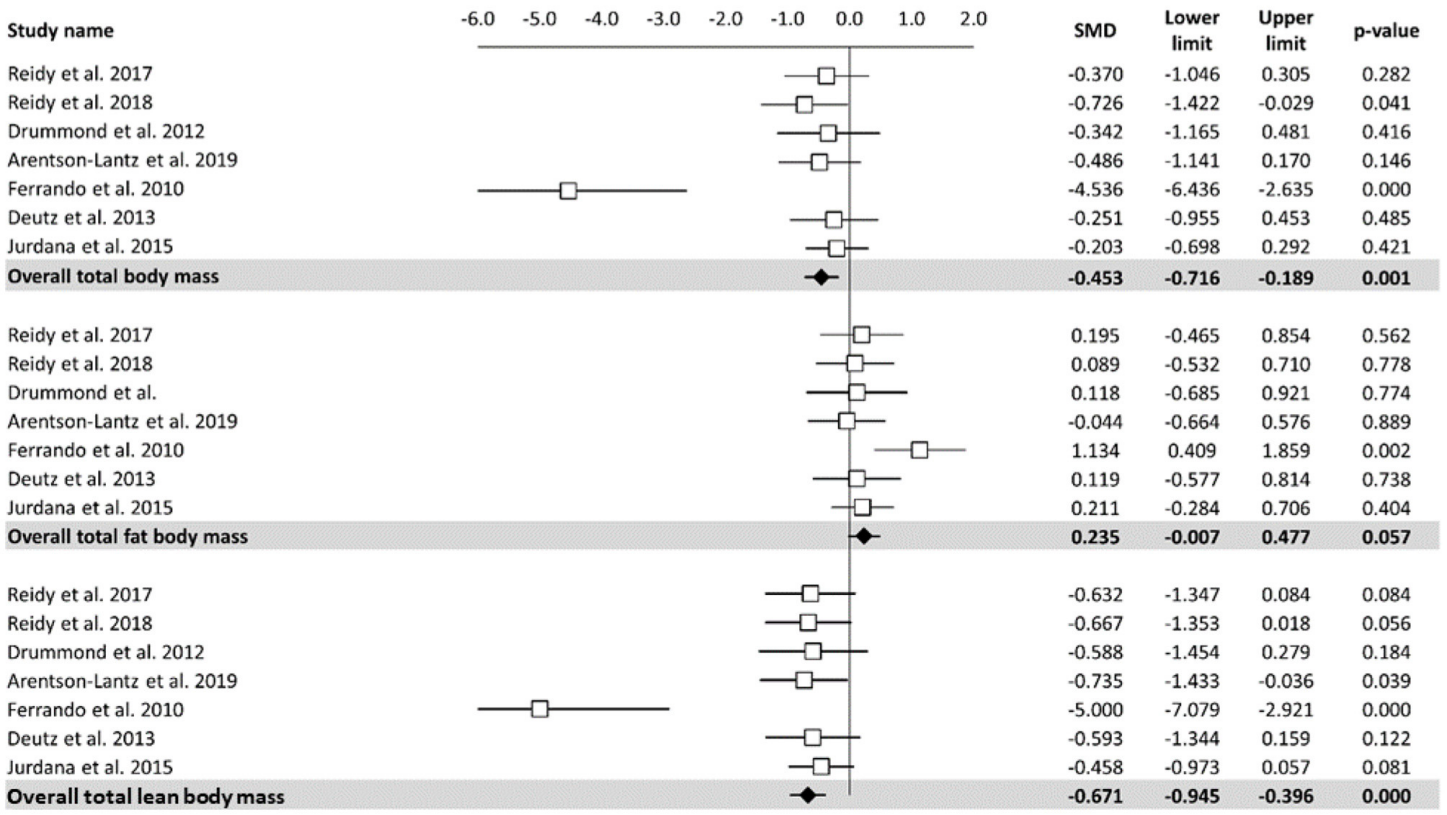
Effects of bed rest on body composition. The results are displayed separately for total, fat, and lean body mass and data expressed as percent differences from the baseline. Mean values and 95% CI are reported for each study as squares and thin line, respectively. Summary results for each parameter are described as solid diamond and thin lines on the gray background. The figures, with *P*-values, are reported on the right part of the plot.

A meta-analysis of the effects of bed rest on body composition is reported in Figure 2. Total body mass was determined as small but statistically significant (ES = −0.45, 95% CI: −0.72 to −0.19, *P* < 0.001). Overall, the total body mass was decreased by 1.2 kg after bed rest interventions. There was no significant relationship between bed rest duration and total body mass (*Z* = −0.913, *P* = 0.361). The heterogeneity of the effect of bed rest on the total body mass was 70%. In contrast, bed rest produced a non-significant effect on the total fat body mass (ES = 0.235, 95% CI: −0.01 to 0.48, *P* = 0.06). Differences in mean values showed that bed rest interventions decreased the total fat body mass by 0.30 kg. Similar to the total body mass, meta-regression (Table 2) did not show a significant relationship between bed rest duration and the total fat body mass (*Z* = 0.648, *P* = 0.517). A low level of heterogeneity was observed for the total body fat mass (*I*^2^ = 15%). Moderate, statistically significant, effects were observed for total lean body mass (ES = −0.67, 95% CI: −0.95 to −0.40, *P* < 0.001) after a bed rest intervention. Overall, the total lean body mass was decreased by 1.5 kg while there was no relationship between bed rest duration and outcomes (*Z* = 0.423, *p* = 672). Heterogeneity among included studies was rated as moderate (*I*^2^ = 65%).

**Table 2:** Meta-regression for bed rest duration and observed outcomes.

	**Coefficient**	**Standard error**	**95% lower CI**	**95% upper CI**	***Z*-value**	***P*-value**
**Body composition**						
Total body mass	−0.040	0.043	−0.124	0.045	−0.913	0.361
Total fat body mass	0.023	0.036	−0.047	0.094	0.648	0.517
Total lean body mass	0.016	0.039	−0.059	0.092	0.423	0.672
**Skeletal muscle performance**						
Knee extension power	0.213	0.050	0.114	0.312	4.219	**<0.001**
Leg muscle mass—old	−0.031	0.046	−0.122	0.060	−0.673	0.501
Leg muscle mass—young	−0.040	0.043	−0.124	0.045	−0.913	0.361

*Bolded values refer to statistical significance of the observed result (p < 0.05), CI, confidence interval*.

#### Effects of Bed Rest on Skeletal Muscle Performance

**Figure 3:**
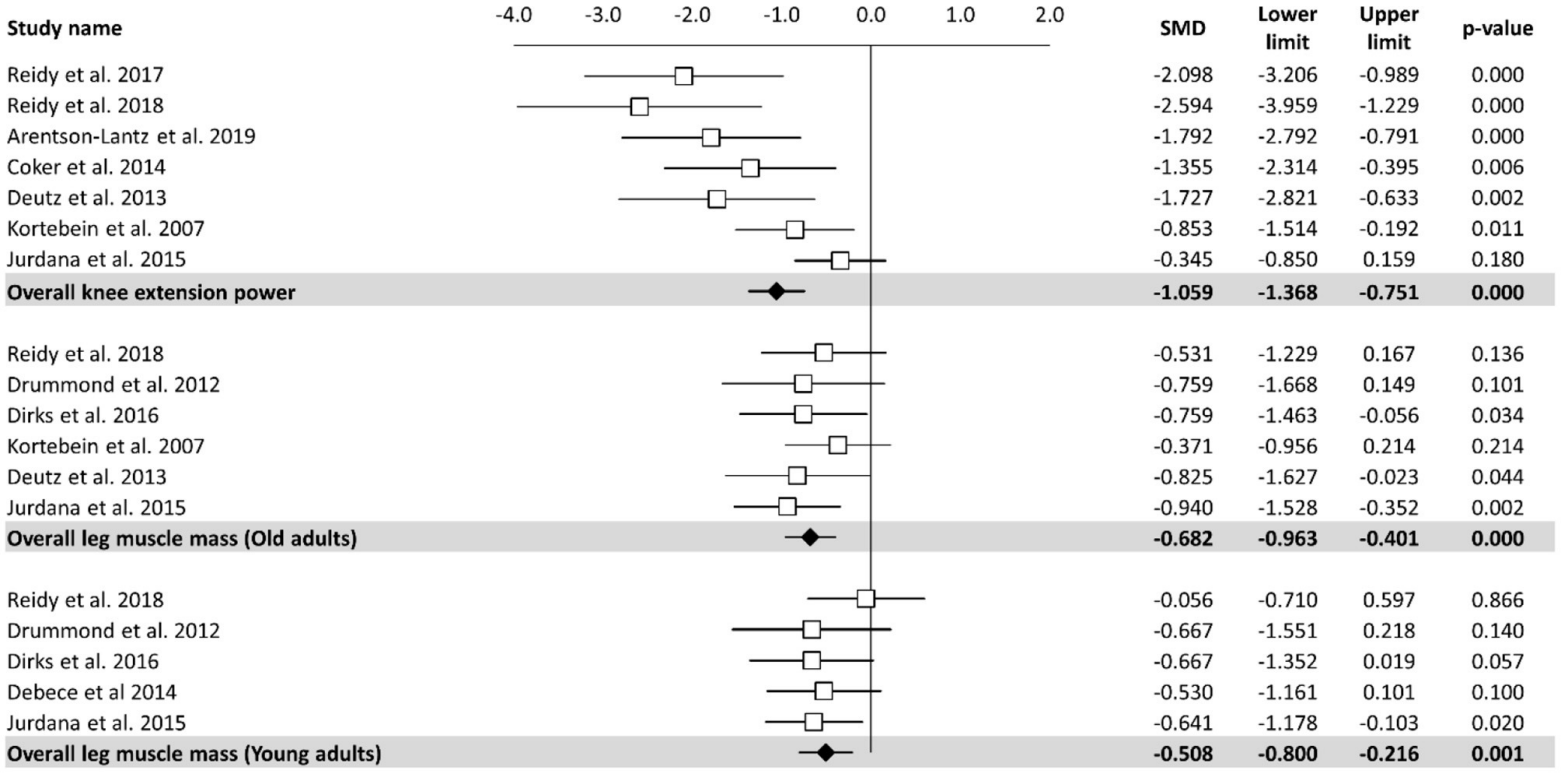
Effects of bed rest on skeletal muscle performance. The results are displayed separately for knee extension power, leg muscle mass in old adults and muscle mass in young adults and data expressed as percent differences from the baseline. Mean values and 95% CI are reported for each study as squares and thin lines, respectively. Summary results for each investigated parameter are described as solid diamond and thin lines on the gray background. The figures, with *P*-values, are reported on the right part of the plot.

Meta-analysis of the effect of bed rest on muscle performance is reported in Figure 3. The meta-analyzed effect showed that bed rest produced a large, statistically significant, effect (ES = −1.06, 95% CI: −1.37 to −0.75, *P* < 0.001) on knee extension power. Knee extension power was decreased by 14.65 N/s. In contrast to other measures, meta- regression (Table 2) showed a significant relationship between bed rest duration and knee extension power (*Z* = 4.219, *p* < 0.001). The heterogeneity of the effect of bed rest on the total body mass was 63%. Moderate, statistically significant, effects were observed after bed rest intervention for leg muscle mass in both old (ES = −0.68, 95% CI: −0.96 to −0.40, *P* < 0.001) and young (ES = −0.51, 95% CI: −0.80 to −0.22, *P* < 0.001) adults. However, the magnitude of change was higher in old (MD = −0.86 kg) compared to young (MD = −0.24 kg) adults. There was no significant relationship between bed rest duration and leg muscle mass in old (*Z* = −0.673, *P* = 0.501) and young (*Z* = −0.913, *P* = 0.361) adults. Heterogeneity was equal to zero for both sub-groups. All 11 trials are at a high risk of bias due to random sequence generation, blinding of participants and personnel, blinding of outcome assessment. In contrast there is no risk of bias in all studies regarding allocation concealment and other bias. Four of twelve studies are at an unclear risk of bias from incomplete outcome data while one of them is at high risk of bias due to selective reporting.

## Discussion

### Effects of Bed Rest

#### Body Composition

Compared to the ambulatory phase, 5 days of BR determine a 2% loss in body mass and a 3% reduction of both total lean mass, as well as of leg lean mass (30–33). Consistently, 7 days of BR induce a −1.6 kg loss (3.2%) of total body lean mass. Notably, 50% of this loss (800 g) came from lower limbs (Pre BR: 18.3 ± 1.1; Post BR: 17.5 ± 1.0 kg). Such a trend is also confirmed after 10 days of BR, with a substantial reduction in total lean mass (~4%) and, to a lesser extent, of whole body mass (−2%) when compared to the baseline. Finally, a similar pattern can be observed also after 14 days of BR with a body mass decrease by 3% and fat-free mass by 5.2%.

The resulting picture is of an early sharp loss of both lean and total body mass within the first 5 days, followed (7–14 days) by a smoother, but persistent reduction in lean body mass associated with a minor loss in whole body mass (Table 3). Several studies demonstrated that changes in fat mass (either loss or deposition) during BR can accelerate muscle atrophy (59, 60). Therefore, eucaloric diet was maintained in almost all studies lasting from 5 to 10 days and has prevented changes in fat mass; consequently, body mass is less influenced by BR as compared to the muscle mass or leg lean mass (Tables 2, 3). Indeed, after 14 days of BR a 5% gain of fat mass has been reported, despite maintenance of a eucaloric diet. Different methodologies for body composition evaluation can play a role in the interpretation of this latter data, with all studies of 5–10-days of BR using Dual Energy X-ray Absorptiometry (DXA) while the 14-day BR study relied on bioimpedance (BIA) technique.

**Table 3:** Time-related bed rest induced body composition relative change (%) in healthy older adults.

**References**	**Participants (n.)**	**Days of bed rest**	**Method**	**Whole-body mass change** **(%)**	**Total fat body mass change** **(%)**	**Total lean body mass change** **(%)**
Reidy et al. (26) Reidy et al. (27)	9	5	DXA	−1.5^*^	0.4	−2.3^*^
Reidy et al. (30) Tanner et al. (29)	10	5	DXA	−2.5^*^	0.4	−4.4^*^
Drummond et al. (30) Drummond et al. (31)	6	7	DXA	−1.8^*^	1.9	−3.2^*^
Arentson-Lantz et al. (32) Arentson-Lantz et al. (33) Arentson-Lantz et al. (34)	10	7	DXA	−1.9^*^	−0.4	−2.8^*^
Ferrando et al. (35)	12	10	DXA	−2.0^*^	0.0	−3.2^*^
Deutz et al. (38) Standley et al. (39) Standley et al. (40)	8	10	DXA	−1.9^*^	1.1	−4.7^*^
Jurdana et al. (43) Pišot et al. (42) Rejc et al. (44) Buso et al. (45) Biolo et al. (41)	16	14	BIA	−3.1^*^	5.0	−5.2^*^

* *All changes from baseline are statistically significant (0.01 < p < 0.05). DXA, Dual energy X-ray Absorptiometry; BIA, Bioimpedance*.

**Figure 4:**
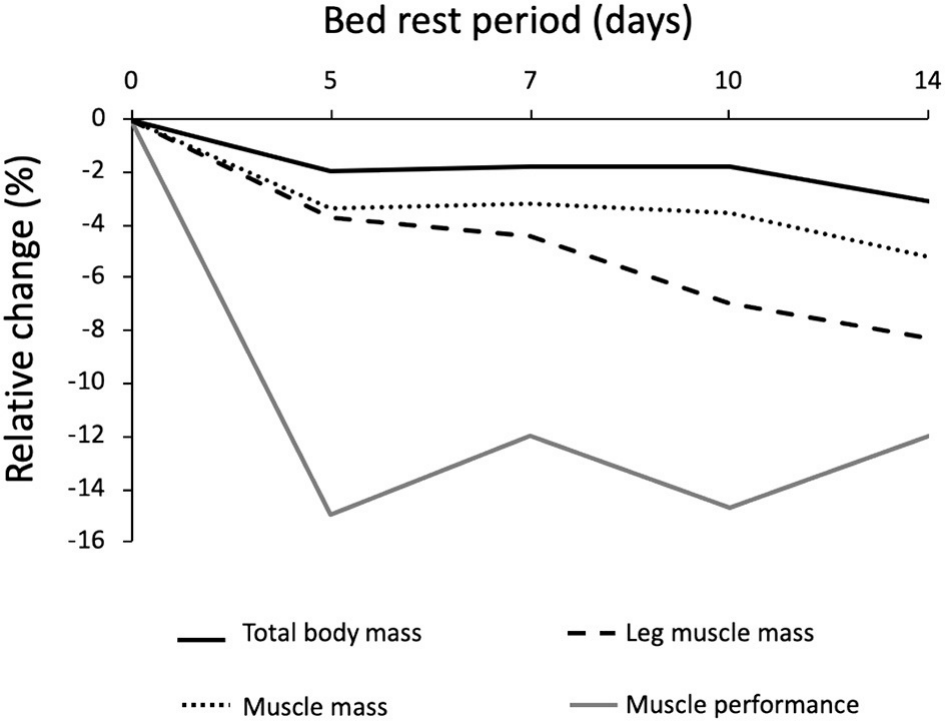
Relative changes of body mass, muscle mass and muscle performance after different periods of bed rest in 59 pooled participants. Data are reported as percent differences from the baseline. Solid black line = total body weight; Dotted black line = muscle mass; dashed black line = leg muscle mass; Solid gray line = muscle efficiency; total *n* = 59 grouped by the duration of bed rest.

Muscle mass and leg lean mass adapt differently to BR (Figure 4). After the first 5 days of BR these two parameters equally declined by around 4%; afterward, leg lean mass continued to decrease by 0.5%/day until reaching −8.5% loss after 14 days of BR, and muscle mass decreased at a much slower rate, reaching −5.5% after 14 days of BR. This discrepancy could at least be partly explained by fluid shifts toward the upper body within the first few days and disproportional body segment usage during the BR where BR induced highest muscle mass declines in those segments with the highest habitual loads before BR (legs).

#### Skeletal Muscle Performance

Evidence indicates that loss of muscular performance (strength and power) often precedes a loss of mass (61). This derangement is probably more important than changes in lean muscle mass alone (62). Indeed, derangement in muscle quality may includes metabolism disorder, loss of aerobic capacity, insulin resistance, fat infiltration, fibrosis, and reduced neural activation, all factors playing a role in muscle efficiency decline (61). These alterations may lead to functional disability (63).

In older adults, different tests have been conducted to evaluate changes in muscle performance following BR. Among them, the two most utilized methods were the Knee Extension Power (KEP), to evaluate muscle power and the Short Physical Performance Battery (SPPB), a group of measures that combines the results of gait speed, chair-stand, and balance tests, as a predictive tool for monitoring muscle function and disability in older people (64). Muscle power is defined as the ability to generate force rapidly (i.e., the product of force and velocity of muscle contraction), while functional capacity is a reliable marker of self-management in daily life living.

The two 5-day BR studies (30–33) showed consistent reduction in KEP of about 14–16% over the experiments (Table 4). This reduction in KEP is confirmed also in strength declines after 7, 10, and 14 days of BR being between 9 and 15% (36–42, 46). SPPB was not reported in the 5 and 7-day BR studies; however, after 10- and 14-day BR SPPB did not change (39–41, 48).

**Table 4:** Time-related bed rest induced delta change (%) in Knee Extension Power of healthy older participants.

**References**	**Participants (n.)**	**Days of bed rest**	**Knee extension power change** **(%)**
Reidy et al. (26) Reidy et al. (27)	10	5	−16^*^
Reidy et al. (30) Tanner et al. (29)	9	5	−14^*^
Arentson-Lantz et al. (32) Arentson-Lantz et al. (33) Arentson-Lantz et al. (34)	10	7	−12^*^
Coker et al. (37)	8	10	−11^*^
Deutz et al. (38) Standley et al. (39) Standley et al. (40)	8	10	−9^*^
Kortebein et al. (23) Kortebein et al. (36)	12	10	−16^*^
Jurdana et al. (43) Pišot et al. (42) Rejc et al. (44) Buso et al. (45) Biolo et al. (41)	16	14	−12^*^

* *All changes from baseline are statistically significant (0.01 < p < 0.05)*.

Besides KEP and SPPB, other tests of functional capacity (i.e., 6-min walking test, timed “up and go” and gait speed, Stair Climbing Power Test) were executed, with a more complex pattern of response to BR. After 5 days of BR, 6-min walking tests, timed “up and go,” and gait speed were unchanged; however, after 10 days of BR, all studies [except one (41)] showed a decrease of 12–14% from the stair-climbing power test (39–42), −7% reduction in 5-mi walk, −8% reduction in walking speed, and a 12% increase in chair-stand time. Although these measures of performance (6-min walk, step-up and go, functional reach, etc.) may not be sensitive enough to detect changes in small groups of volunteers following very short BR periods (61), significant changes were identified after 10 days of BR. They were consistent with studies in bed-ridden hospitalized elders demonstrating a clear negative relationship between BR and functional capacity (23, 26, 65). The association between the amount of time spent in bed at home and the extent of functional decline in social, instrumental, domestic, and physical activities were also observed in a community setting over a time period of 18 months (24).

**Figure 5:**
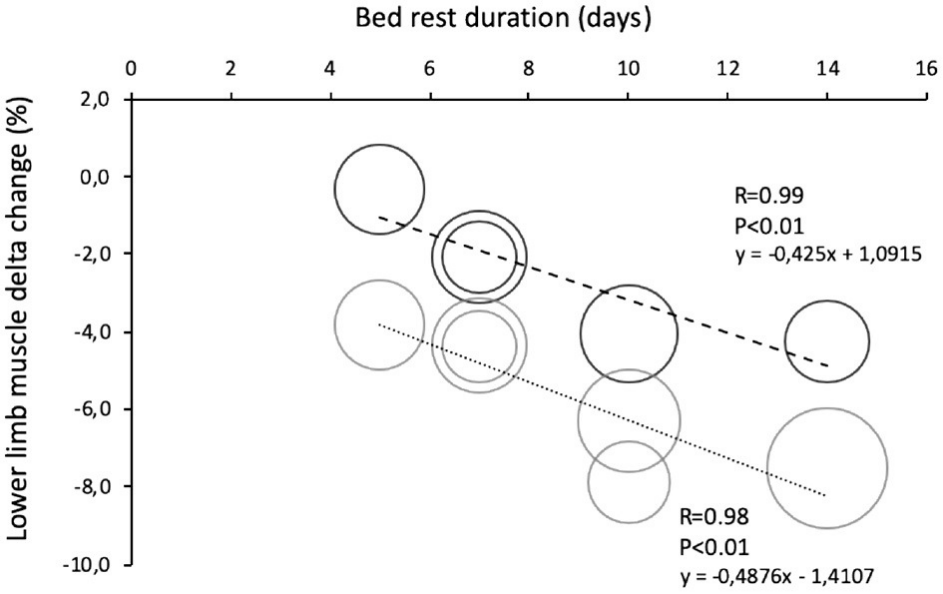
Comparison of lower limb muscle change in young and old during bed rests of different duration. Data are reported as percent differences from a baseline. Solid black round = muscle leg loss in healthy young volunteers (total n = 46 grouped by duration of bed rest). Solid gray round = muscle leg loss in healthy elderly volunteers (total *n* = 59 grouped by duration of bed rest).

The loss of muscle performance, followed a different pattern when compared to the muscle mass decline (Figure 5). Muscle performance showed a reduction of 3%/day during the first 5 days of BR and then stabilizes until the 14th day of BR, reaching −13%. These latter results are consistent with several other cross-sectional studies in ambulatory conditions, reporting a disproportionate decrease in muscle mass and strength in elderly participants (66–70). The discrepancy between the loss of muscle quantity and function in <14 days of BR must be further explained by a decrease in “muscle quality,” tendon alterations, and neural factors: muscle architecture change (71), reduced neural drive (72, 73), muscle denervation and NMJ damage (10, 74, 75), alterations in tendon mechanical properties (74), muscle fiber atrophy and force reduction (76), and more. Another possibility is that the physical performance tests are not sensitive enough for changes observed during BR. We can also speculate that skeletal muscle anabolic compensatory pathways require some days to be activated, or that some breakdown/remodeling ones have a time dependent regulation, with broad-spectrum breakdown taking place in the first days, followed by a more selective degradation (possibly ubiquitin-proteasome mediated) toward less active/effective fibers. The actual available literature does not provide serial measurements of these parameters.

Skeletal muscle cross-sectional area and myosin heavy chain myofibers composition. A direct comparison among the different studies (14, 30, 33, 36, 44, 46, 48) must consider some methodological issues, such as manual vs. computerized reading of the CSA areas, the assessment of potential pre-BR physical activity, or sport training, and intrinsic variability in CSA within the same muscle. This leads to highly variable results in the CSA which are somehow discussed: not all papers report the absolute data and when absolute values are shown, CSA variability is described as the standard error (which, given the number of observations, is typically one-third of the standard deviation). Such a variability, higher than that observed in other markers, probably reflects intrinsic heterogeneity within the same muscle (77) and questions the parametric statistical approach used in these studies.

Two studies reported a ~20% decrease in CSA after 5-days of BR (30, 31, 33). Similarly, a 15% decrease in CSA was observed after 14 days BR, in middle-aged (14) and older adults (46, 48). Oddly, in the 7- and 10-day BR studies, no difference from baseline could be observed, with even a trend toward an increase in CSA (27, 36–44). It is impossible to determine whether these conflicting results are related to differences in population type (active vs. non-active) and size, in protocol design (recruitment or selection), or on the above-mentioned methodological issues. Similar contrasting results can be observed also for myosin heavy chain myofibers (MHC) composition changes. After 5 days of BR elderly adults showed a strong decrease in myofibers of vastus lateralis, mostly driven largely by MHC I fiber types (30, 31, 33). Nonetheless, after 10 and 14 days of BR, no significant changes were observed (43, 44, 48).

### Comparison of Younger and Older Participants

#### Body Composition, Muscle Performance, and Muscle CSA/Volume

The comparison between elderly and young participants is useful in evaluating the combined effects of the age groups and BR duration on muscle mass and function. So far, only three BR studies (lasting 5 and 14 days) report a direct comparison between young and elderly participants (32, 33, 46). The missing comparisons (at 7 and 10 days) were filled from matching data from BR performed only on elderly participants (27, 34, 35, 40, 41) and matching data from BR performed only in young participants (78, 79). It is known that BR mostly affects lower limb muscles, thus leg lean mass changes are considered a better index in estimating muscle mass loss during BR. In the two 5-day BR studies, leg lean mass decreased only in old (−3.6%) as compared to young (−0.3%). In longer BR studies we found a linear trend in decreased lower limb muscle mass with the BR duration (Figure 5). Specifically, data from 7-days of BR show that leg lean mass decreased more in older (34, 35) than in younger participants (78, 79) by −4.4 and −3.1%, respectively. That was confirmed also after 10 days of BR when the decline in older participants was 6% (22, 40, 42) and in young 4% (78) as well as after 14 days of BR, when younger participants declined by 6.1% and older participants by 8.3% (46). As expected, a eucaloric diet that was incorporated in all reported studies must have prevented higher muscle declines (59, 60) and also significant body fat mass changed both in young and older participants. Data from BR studies reported a non-significant body fat variation between 0 and 5% without interaction, of an age group of participants (30–34, 36–38, 43, 44, 46–48, 61, 78, 79).

Age-related differences in muscle volume have been investigated in both young and old volunteers after 5 and 14 days of BR (30, 31, 46). Quadriceps CSA declined only in older subjects after 5 days of BR by −3%, while quadriceps volume continued to decline up to 14 days of BR by 8.3%. Such a reduction being more important in older than in younger counterparts, that declined by 6.1%. It should be noted that, in both studies, younger participants had 11 and 23% higher baseline muscle volume and CSA than older ones, respectively. One of the most important triggers of muscle decline during inactivity could be resistance to post-prandial anabolic stimulation of protein synthesis. Biolo et al. (45) demonstrate that anabolic resistance induced by experimental bed rest is much greater in the elderly, as compared to a younger population.

It is also evident that muscle performance deteriorates more in older than in younger volunteers. Specifically, 5 days of BR lowered leg strength more in older (12%) than in younger (9%) (32, 33) with a significant interaction effect. After 14 days of BR, two parameters expressing the contractile function (maximal voluntary isometric force and lower body explosive power) declined only in old for 13 and 15.2%, respectively (46). Both parameters show a declining trend also in younger subjects (11% for both parameters), although these differences were not significant (*p* < 0.100) due to the small size (*N* = 7) of the population.

### Effect of Rehabilitation

#### Body Composition Muscle Efficiency and Cross-Sectional Area

Only three BR studies reported results of a rehabilitation program. Reidy et al. (33) and Tanner et al. (32) combined a 3 week exercise rehabilitation protocol with a BCAA enriched whey protein supplement to recover from the effects of 5 days of BR. Authors found that, independent of age group, rehabilitation restored leg lean mass (Young: + 2.63 ± 1.09%; Old: + 3.78 ± 1.40%) and KEP (Young: 42.66 ± 11.05%; Old: 25.16 ± 6.67%). As for LEU supplementation (34), even if it confers a moderate protective effect on lean leg mass during 10 days of BR, following rehabilitation, leg lean mass in both intervention and control groups returned to the baseline values.

In the second study, Deutz et al. (42) and Standley et al. (43, 44) prescribed 8 weeks of rehabilitation to all participants (both controls and HMB supplementation group). After rehabilitation, muscle mass gain did not differ between the two groups. However, the HMB group showed an improvement in KEP from the baseline (+11.15 Nm/s, *p* = 0.03) while the control subjects only regained their baseline level (+5.90 Nm/s, *p* = 0.4). Moreover, the HMB group showed an increase in muscle functionality (Timed Get-Up-&-Go) during the rehabilitation period that was not evident in the control group.

In the third study (46), 14-days of BR were followed by 2 weeks of supervised, multimodal rehabilitation exercise program in young and old volunteers. Rehabilitation increased body mass more in young than in old (Old: +2.4; Young: +3.9%; *p* < 0.001). However, only older participants showed an increase in total body fat-free mass (4.4%, *P* < 0.006) and quadriceps muscle volume (5.7%, *p* < 0.001). Notably, rehabilitation did not restore the baseline muscle volume in older subjects (−3.1%, *p* = 0.048) but it did in younger ones (−1.7%, *p* = 0.428). It is also important to remember that, after 14 days of BR, knee extension maximal voluntary isometric force and maximal explosive power declined only in old people; however only knee extension force recovered completely after the rehabilitation.

As for CSA, the rehabilitation protocols seem more effective (+42%) in the elderly after 5 days of BR, compared to the younger volunteers, who show no effects. By increasing the BR period, such effects seems less obvious within each comparison (slow vs. fast fibers, young vs. old participants).

### Muscle Protein Kinetics

Physio-pathological conditions (e.g., sarcopenia of aging and disuse) are characterized by reduced efficacy of an anabolic agent to stimulate protein synthesis. This condition, commonly defined as anabolic resistance, is considered a mechanism contributing to muscle atrophy following prolonged physical inactivity (80–82). Therefore, evaluation of the skeletal muscle protein turnover is critical to assess the negative effects of BR, its mechanism(s), and the efficacy of possible countermeasures and/or rehabilitation programs. Anabolic resistance is evaluated through complex and invasive methodologies based on intravenous infusion of stable isotopes of amino acids (76, 83). Only three studies explored bed rest-induced changes in protein kinetics.

Drummond et al. (34) showed that, in ambulatory conditions, elderly muscle protein synthesis rate increased by 40% following acute ingestion of EAA, but not after 7 days of BR. Consistently, Ferrando et al. (39) found that 10 days of BR determined a 30% decrease in muscle protein fractional synthesis rate in healthy elderly volunteers, but not in subjects having a daily supplementation of EAA. Finally, Biolo et al. (45) showed that 2 weeks of BR reduce post- prandial protein kinetics more in elderly participants (−33%) than in the younger (−11%) adults. Unfortunately, the resulting data was not comparable due to the different methodologies applied. Nonetheless, all three studies corroborate the concept of anabolic resistance in elderly bedridden volunteers.

## Conclusion

Experimental BR is a suitable model to study detrimental effects on body composition, physical performance, and biochemical changes induced by inactivity in young, old, and hospitalized people. This experimental approach allows for splitting of the intrinsic effects of skeletal muscle inactivity from the disease-related changes in structure, activity, and pathophysiology.

The different studies show a remarkable consistency for some investigated variables, particularly considering the different duration of the protocols. Changes in muscle mass and function are the two most investigated variables and allow for a consistent trend of the BR-induced changes. It remains to be seen the exact mechanisms of the discrepancy between muscle mass and performance decline after BR.

Facing a BR period, young and elderly adults react differently. Older adults lose more promptly muscle mass than younger, while muscle efficiency declines only in older adults at 14 days of BR. Countermeasures during BR and rehabilitation after BR have different efficacy in muscle mass and strength maintenance and/or recovery. Apparently, all the nutritional and physical (i.e., NMES and 2000 steps/day) countermeasures carried out during BR are not fully effective in blunting muscle mass loss, although they seem able to counteract the inactivity-related anabolic resistance. On the contrary, all the rehabilitation programs conducted (from 2 to 8 weeks) after BR, restored (in older adults) or even improved (in younger adults) the pre-test levels of muscle mass and function. All these studies are important to corroborate early clinical evidence that muscle loss in old age is more rapid during the early stages of hospitalization (52) and highlight the importance of countermeasures combined with a rehabilitation protocol to promote complete recovery afterward.

Further BR studies are needed to investigate the mechanism and comparative time course of the processes occurring in muscle reduction during BR in the elderly to fully understand discrepancies between the age groups, as well as the link between muscle mass and performance declines.

## Data Availability Statement

The original contributions presented in the study are included in the article/supplementary material, further inquiries can be directed to the corresponding author.

## Author Contributions

FD and GB research design, data analysis and statistical analysis, manuscript draft and had primary responsibility for final content. NF, BS, and ZM data analysis and interpretation, statistical analysis, manuscript draft. MN and RP research design, manuscript draft. FM, PV, and RS manuscript draft. All authors have read and approved the final manuscript.

## Conflict of Interest

The authors declare that the research was conducted in the absence of any commercial or financial relationships that could be construed as a potential conflict of interest.

## Publisher's Note

All claims expressed in this article are solely those of the authors and do not necessarily represent those of their affiliated organizations, or those of the publisher, the editors and the reviewers. Any product that may be evaluated in this article, or claim that may be made by its manufacturer, is not guaranteed or endorsed by the publisher.
